# “Mars Bar and a Tin of Red Bull Kept Me and My Patients Alive”: Exploring Barriers to Healthy Eating through Facebook Comments of Shiftworkers

**DOI:** 10.3390/nu15040959

**Published:** 2023-02-15

**Authors:** Emma McIntosh, Sally A. Ferguson, Jillian Dorrian, Alison M. Coates, Gloria Leung, Charlotte C. Gupta

**Affiliations:** 1 Appleton Institute, School of Health, Medical, and Applied Sciences, Central Queensland University, Adelaide 5034, Australia; 2 UniSA Justice and Society, University of South Australia, Adelaide 5072, Australia; 3 Alliance for Research in Exercise, Nutrition and Activity (ARENA) Research Centre, University of South Australia, Adelaide 5000, Australia; 4 Department of Nutrition, Dietetics and Food, Monash University, Notting Hill 3168, Australia

**Keywords:** shiftwork, night shift, motivations, food choice, Facebook, time-restricted eating

## Abstract

The negative impact of an unhealthy diet on the shiftworker population has been well-documented. However, little evidence exists on the underlying reasons for unhealthy eating behaviours and the existing barriers to healthy eating withinshiftwork environments. This qualitative study investigated the dietary behaviours reported by shiftworkers through Facebook comments. Comments were collected if they were on public shiftworker-relevant posts pertaining to dietary news or dietary information on Facebook and were posted by self-identified shiftworkers, relatives of shiftworkers, or partners of shiftworkers. A thematic analysis of the 144 comments collected generated four categories that can be used to understand the motivations for eating behaviour on-shift: what shiftworkers eat, where food is sourced from, when food is eaten, and why certain foods are chosen. Results reveal motivations, attitudes, and both internal and external barriers to healthy eating behaviours, as well as similarities and differences across shiftwork industries. Recommendations for future research include further explorations on the link between scheduled eating (e.g., time-restricted eating) and shiftwork, the impact of a rotating shift arrangements on dietary health behaviours, and the impact of interpersonal relationships on shiftworker dietary choices. Understanding these motivations will inform strategies to promote healthy eating and help understand barriers for shiftworkers.

## 1. Introduction

Shiftwork refers to work arrangements outside of typical daytime working hours, with shiftworkers providing essential services 24 h a day [1]. In Australia, the United States of America, and the United Kingdom, shiftwork arrangements are most commonly required in healthcare and social assistance, accommodation and food services, and mining industries [2,3]. Further, while most shiftworkers return to their homes to sleep between shifts, another shiftwork arrangement involves fly-in fly-out work (FIFO). This is specific to places of work of significant isolation [4] and involves employees travelling to and from their worksite and living in the provided accommodation during their roster, commonly for one to four consecutive weeks [5].

Common amongst the different shiftwork industries are the benefits of shiftwork such as increased pay or compensation [6] and flexibility in attending to family/home life commitments [7]. However, also common, are concerns about the impact of shiftwork on health. Compared to the general population, shiftworkers have a greater risk of several short- and long-term health consequences. Shiftwork has been linked to increased levels of overall stress [8,9,10,11,12], poor sleep quality [1,13,14,15,16], heightened risk of cardiovascular disease [1,17,18], and an increased risk of obesity [19,20]. Notably, these findings can be demonstrated across various shiftwork industries [18].

A healthy diet, incorporating both the quality and quantity of food, plays a major role in the health of shiftworkers [21,22]. As part of a healthy diet, public health policy advocates for a balanced diet consisting of a variety of nutritious foods such as fruits, vegetables, legumes, nuts and wholegrains, with minimal consumption of free sugars, processed foods, and saturated fats [23,24]. This healthy diet has been shown to be key for health outcomes including a lowered risk of mortality, cardiovascular disease, and cancer [25]. However, many shiftworkers experience barriers maintaining these healthy eating behaviours, such as environmental, biological, and social factors [21,26,27]. For example, previous research has demonstrated factors such as long working hours, a lack of breaks [28], the lack of availability of healthy food during night shifts [26], and stress [29,30] as major barriers to making healthy choices.

In addition to the quality and quantity of food, timing of eating has emerged as another major factor influencing the health of shiftworkers [22,31]. For optimal health, research suggests several key aspects of good eating patterns including a consistent daily eating duration (that is, the time between first and last meal) of fewer than 12 h per day, consuming the majority of kilojoules in the earlier part of the day, and avoiding food intake close to bedtime or during the night [32]. These regular eating patterns are associated with good health outcomes, including a lowered risk of cardiovascular disease and obesity [22,31,32]. However, in addition to challenges in maintaining a healthy diet, shiftwork presents challenges to maintaining regular eating patterns [21,26]. Shift timing leads to changes in a shift worker’s sleep/wake cycle, and these changes can disrupt typical diurnal eating patterns. For example, after a nightshift, a shiftworker is likely to sleep during the day, therefore missing out on one or more of the typical diurnal mealtimes (i.e., breakfast, lunch, and/or dinner). When these diurnal mealtimes are missed, shiftworkers often then redistribute food across the 24 h period, and this may include eating during the night [26,33,34,35,36].

Taken together, the factors that influence the healthy diet and eating patterns of shiftworkers can be considered the ‘what’, ‘when’, ‘why’ and ‘where’ of shiftworker eating behaviour [26]. Therefore, to improve the health of shiftworkers, targeted guidelines and recommendations for healthy eating practices (incorporating the type and timing of food) are needed [21,26]. This requires a thorough understanding of the current barriers to healthy eating that exist in shiftwork settings. To date, the literature has investigated these barriers through questionnaires, interviews, dietary recalls, food diaries, and focus groups [26]. However, it is known that the validity of dietary data can be influenced by social desirability bias, whereby participants selectively report dietary behaviours due to concern over judgement from a researcher or nutritional professional [37]. Therefore, to further the current findings and ensure future research is representative of actual nutrition behaviour, alternate methods of data collection should be considered.

Social media interactions via posts and discussion threads have been utilised in the wider literature to understand perceptions and decisions on health topics [38]. “Digital ethnography” has been used in recent literature to describe the study of people in a real-world environment, such as Facebook [39]. The use of social media for research purposes provides an opportunity to represent otherwise inaccessible participants, on an international level [40]. Due to the accessibility of self-reported data, Greene et al. [41] found Facebook was a source to share personal clinical information, guidance on disease-specific concerns, and support. As of July 2021, there is an average of 1.91 billion daily active users on Facebook, making it one of the most dominant social media platforms [42]. As a result, there are many opportunities to explore and understand the dietary behaviours of shiftworkers from a diverse range of industries, backgrounds, shift-types, and with differing levels of experience. Further, it is likely that Facebook posts are less influenced by the social-desirability bias that can impact how honest or forthcoming participants are when reporting dietary habits to researchers [37], as Facebook users are not in a research setting, and are choosing to publicly share their behaviours or opinions with other members of the public.

This study aimed to explore barriers to the healthy eating behaviours of shiftworkers through Facebook comments. Investigating dietary behaviours among shiftworkers through comments on Facebook, an information-rich social media platform, provides unfiltered and potentially novel information that may not have been captured in previous literature. Additionally, as Facebook is a global platform and freely available for users, data can be captured from shiftworkers from a variety of industries and locations. 

## 2. Materials and Methods

### 2.1. Study Design

This qualitative study received ethics approval from the Central Queensland University Human Research Ethics Committee (2021-099).

### 2.2. Participants and Recruitment

As this was an exploratory study, to gain a broad perspective on eating behaviours and barriers to healthy eating, participants were anyone who commented on the eating patterns of shiftworkers, and this included self-identified shiftworkers in addition to relatives of shiftworkers, and partners of shiftworkers. These comments were accessed through public pages and public groups specifically relating to shiftwork. No identifying information was collected and therefore multiple comments may have been included from a single user. As this was an exploratory study, the inclusion criteria were broad and included any comment that referenced shiftwork shiftwork and eating patterns and behaviours. Exclusion criteria were based on the content of the comments: (1) contains specific mentions of other Facebook user/s, (2) advertisements, and (3) unrelated links and content. Only comments in English were considered for analysis. 

### 2.3. Procedure

The Facebook search function was used to explore comments in public pages, public posts and public groups relating to shiftworkers and their opinions and experiences with eating behaviours and eating patterns. The searches were started in October 2021. Data were collected until data saturation was reached, which occurred in November 2021 [43]. Data saturation was determined to be when no new codes emerged, and the same public pages and public groups were shown in the Facebook search results. The keywords used in the Facebook search function included variations of “shift work”, “nutrition”, “diet”, “wellbeing”, “food”, along with key shiftwork industries representative of the Australia and New Zealand Standard Industrial Classification [44]. The comments were in response to posts about shiftwork and eating, such as news stories, research articles, dietary questions, and newspaper articles that prompted a discussion in the comments section of eating behaviours and shiftwork. The de-identified comments were manually copied from Facebook and pasted into a spreadsheet. Due to the nature of the study, the data collection procedure was performed without interaction with Facebook users. 

### 2.4. Data Analysis

Comments were coded and analysed by author EM and reviewed by CCG using thematic content analysis procedures, using NVivo (Version 13). Using a deductive process, comments that discussed a barrier to eating were grouped into the following categories: what type of foods shiftworkers eat, when shiftworkers eat, where food is sourced from, and why shiftworkers choose to eat on shift. These categories are based on a previous review by Gupta et al. [26] that synthesized the literature to identify motivations for eating in shiftworkers, and subsequently identified what, when, where, and why as the key factors influencing eating. Following this process, we used an inductive process within each category (what, when, where, why) to identify themes, with the aim of gaining a more in-depth understanding of how the categories are operationalized. Information was also collected from the comments on the shiftworkers’ working arrangement or industry role to describe the shiftworkers represented. All percentages presented in the results (including the percentage of comments in each theme, and from different shiftworking industries) were rounded to the nearest whole decimal.

## 3. Results

### 3.1. Characteristics

The identified Facebook posts were published between September 2014 to October 2021, and included news stories about shiftwork-related research, blog posts about shiftwork and eating, posts by shiftworkers asking for tips on eating, and articles about specific eating strategies, such as intermittent fasting. 144 distinct comments were identified that adhered to the inclusion and exclusion criteria. Among the comments, 17% (24/144) were written by shiftworkers that self-identified as practicing nurses or retired nurses, 10% (14/144) identified as a truck driver or working within the truck driving industry, 9% (13/144) of comments were identified as FIFO workers and 4% (6/144) were partners of FIFO workers. Overall, 41% (59/144) identified as night shiftworkers.

### 3.2. Overview of Themes 

**Figure 1:**
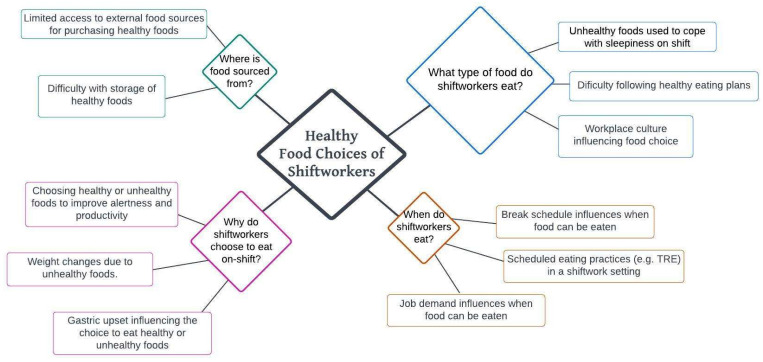
The four categories of the comments and the themes identified within each category. Theme diamonds are sized according to the number of comments coded to this, with a larger diamond representing a larger percentage of comments on this theme, as a percentage of total comments. Each comment could be coded to more than one theme.

Thematic analysis revealed four themes, see Table 1. A majority of comments (51%) were in the category “what type of foods do shiftworkers eat” and the themes of this category were the work environment influences the type and quantity of food, (Figure 1). 

### 3.3. What Type of Foods Do Shiftworkers Eat?

The majority of comments in this category described coping with sleepiness as a motivation for food choice on-shift and a barrier to healthy food choice. Sleepiness was a common influence for choosing unhealthy and highly processed snacks, with increased sleepiness discussed in relation to overeating to improve alertness and reduce sleepiness.

“0400 Mars Bar and a tin of Red Bull kept me and my patients alive”

In some comments, shiftworkers reported the drive home from a shift was a difficult experience due to feelings of sleepiness and this influenced what they chose to eat. Several comments identified various foods kept in their car to sustain energy levels, for example, confectionery and fruit cups.

“Eat some cut up fruit on the drive home to keep me awake.”

An additional theme that emerged was healthy or unhealthy food choice based on workplace culture, with eating food on-shift providing an opportunity for socializing and team bonding. Comments specifically reported this within the nursing industry and among FIFO workers. Shiftworkers in the nursing community recalled experiences of social eating practices during working hours, where unhealthy snacks were shared amongst colleagues, such as chocolate and cake. These social occasions were described as an enjoyable aspect of going to work, but also motivated them to make unhealthy dietary choices. Likewise, a notable pattern among FIFO worker culture were tendencies to choose large portions of unhealthy meals. Due to the buffet-style food services on-site, subjects noticed themselves and their colleagues were motivated to choose large amounts of food for each meal, choosing to consume multiple plates of food, and selecting unnecessary confectionery items. As a collective, the content of the comments expressed a strong level of enjoyment associated with engaging with workplace food culture and bonding with colleagues by making similar food choices. 

The third theme that emerged in this category was difficulty following specific diets when working shiftwork, with this then influencing what can be eaten on-shift. Comments from shiftworkers suggested that the work environment made it difficult to follow specific diets such as vegetarianism, or plant-based diets.

“Humans weren’t meant to do this kind of work schedule, and it’s difficult to do so on a plant based diet. I still haven’t figured out how to do it without leaning on vegan processed foods”

### 3.4. Theme 2: Where Is Food Sourced from?

A theme that emerged from this category was the limited access to external food sources which was a barrier to healthy food choices. Among the nursing community, the availability of food to purchase was dependent on the time-of-day people were completing a shift. The comments highlighted difficulties accessing food, particularly healthy foods, within the hospital environment, which included vending machines, hospital cafeterias and takeaway restaurants. Depending on shift type, hospital cafeterias were described as not providing the appropriate meal for that time of the shift (e.g., breakfast, lunch and dinner) and largely providing processed foods. In this way, this food source was a barrier to consuming healthy food on-shift. Further, opening hours of both cafeterias and takeaway venues did not always cover night shifts. Comments noted that, when faced with overtime, an unplanned double shift or the inability to take a break, the only options were vending machines or the hospital cafeteria. However, vending machines were identified as a major concern due to a surplus of food that are high in fat, sugar and salt.

“It’s time the cafe situation at hospitals was fixed. Limited healthy food and sky high prices.”

Comments from workers in the road transport industry also identified a limited access to food sources. Comments posted in response to the dietary standards of truck drivers emphasized a high reliance on roadhouses (service stations) and fast-food restaurants to provide meals.

“How do they expect you to eat healthy on the road when all there is to eat is dirty stinking Mc Donald’s sometimes”

An additional theme that emerged was difficulty sourcing healthy options. Comments from nurses described being able to purchase meals from supermarkets for a healthier option on-shift, however a barrier to this was whether they had access to cooking and storage facilities, such as a fridge or microwave. Further, FIFO workers described the type of food supplied by the catering services on-site as unhealthy and having a limited variety of foods, particularly for night shiftworkers. These unhealthy meals were partly attributed to the quality of the produce, standard of cooking, and presentation. As a result, comments did describe sourcing food from a supermarket or bringing additional long shelf-life foods and supplements on-shift to support their dietary needs.

“My husband and his crew stay at a pub and most food is deep fried and lunches are pies & pasties. He is noticing all the men increasing in size including himself.”

Difficulties with food storage also influenced where food was sourced from, with comments highlighting barriers to bringing healthy food options from home onto shift. Among the comments, 38% described bringing healthy food items such as fish, vegetables, fruits, yoghurt, nuts, and seeds onto shift. However, these comments were largely from doctors and nurses, with comments from the mining industry describing limited access to food storage and preparation facilities (e.g., fridge space), which influenced what food they ate on-shift.

“That’s all well & good if your [sic] on camp however when you’re in the cheapest motel available with no fridge or basic facilities it’s very difficult!”

### 3.5. Theme 3: When Do Shiftworkers Eat?

Within this category, break schedule emerged as a main barrier to eating on-shift and maintaining healthy eating patterns. Comments described shiftwork as an unpredictable and demanding job and, as s a result, break times largely dictated when shiftworkers could eat on-shift. A lack of break corresponded to a lack of food.

“A lot of our emergency services do not even get a meal break due to them being so busy. Sometimes their only choice is to scoff a macca’s cheese burger or chocolate bar on the way to their next job”

Interestingly, timing of breaks was not a barrier to healthy eating for some shiftworkers, with comments reported a benefit to structuring eating timing around breaks, and a struggle with not having set breaks when off-shift.

“I find it easier to eat healthy when on shift. Your breaks are the same every night, you know you have to eat. I struggle on my days off to eat enough/properly”

A further theme was the impact of job demand on-shift, with a comment from a spouse of a shiftworker describing job duty and responsibility on-shift as impacting what their husband ate and acting as a barrier to healthy eating on-shift.

“While I do my best to cook my husband healthy home cooked meals for work, sometimes those meals end up left in the work fridge or in the bin because everyone else’s safety comes before his dinner”

Scheduled eating strategies also emerged as a theme within this category, with two types of scheduled eating discussed, Time-Restricted Eating (TRE) and Intermittent Fasting (IF). Comments posted in response to scheduled eating techniques provided hour-by-hour plans on when subjects chose to eat and fast during a working day. Additionally, several subjects commented that TRE and IF would not align with a shiftwork schedule (or would be inappropriate for shiftwork). Namely, comments suggested that a lack of energy and high work demands would impact the ability to adhere to an eating routine.

“[about time-restricted eating] very doable if you are 9 to 5, but it isn’t very helpful when you work shifts. Maybe there could be some suggestions directed to people who have to do shifts to help them keep their body repairing itself properly.”

However, some comments from workers who managed to integrate scheduled eating into their working day described positive outcomes, such as a reduction in weight and an overall feeling of improved health.

“I eat between 5 p.m. and 7 p.m. and then fast for 22 h essentially-I feel SO MUCH BETTER!!”

### 3.6. Theme 4: Why Do Shiftworkers Choose to Eat On-Shift? 

Increasing alertness and productivity emerged as a theme influencing the motivation to eat on-shift. Comments from workers in the healthcare industry described eating to improve alertness and that this impacted their work performance.

“I eat every 3–4 h but healthy choices and the odd time I find myself slipping I notice a huge difference not only in how I feel but my productivity decreases drastically”

Further, the need for sleep post-shift emerged as a theme influencing the choice to eat on-shift. This theme was present in several comments, especially among the nursing community. Food choices on-shift were influenced by the perceived impacts of foods on sleep post-shift, with certain foods avoided.

“I find that if I don’t have any coffee or junk foods, sugar etc I don’t get the post night shift hang over and I sleep better during the day”

An additional theme emerging was how weight changes influenced the choice to eat on-shift. Weight gain and weight loss were commonly viewed by shiftworkers as an indicator of whether they were following healthy eating patterns. Weight gain was consistently regarded as an undesirable effect of working unconventional hours. As a result, comments described various constructive efforts to prevent weight gain, such as avoiding unhealthy food when on shift.

“I too put on a few kg’s when I started but that’s because I ate all the crisp foods and always had desserts.”

Strategies for coping with gastric upset on-shift also emerged as a theme explaining why shiftworkers may choose to eat on-shift. Several comments discussed how to avoid experiencing various digestive issues, including gastrointestinal inflammation and indigestion. Strategies included restricting the amount of food consumed during a shift, either to abstain or adhering to a scheduled eating routine. Other comments discussed the use of natural remedies.

“Bread and pasta give me a bloated tummy overnight. I stick to nuts, fruit and yoghurt”

## 4. Discussion

This study was the first to examine elements of the eating behaviours of shiftworkers through Facebook comments. We identified key themes that described what food shiftworkers choose to eat on-shift, where food is sourced from, when shiftworkers eat on-shift, and why shiftworkers choose to eat on-shift. 

Critically, the novel methodology used in this study allowed us to identify similar experiences of shiftworkers across multiple industries and shift-patterns using comments from shiftworkers and partners of shiftworkers While the influences of shiftwork on the food choices of nurses, FIFO workers, and truck drivers have been explored previously [45,46,47], this is the first study to highlight that shift workers across different industries experience similar influences and eating behaviours. This highlights that despite different job demands and responsibilities on-shift, different shiftwork industries face many similar barriers to healthy eating. Additionally, the health effects of eating behaviours on-shift were common across industries, with the most common effects in the comments including changes in weight, effects on sleep, and stomach upset. Comments demonstrated that these effects influenced the decisions made about what foods were eaten, and at what times, reflecting reports in the literature [21,26,36]. Taken together, similar results across industries supports the need for general shiftwork eating guidelines that can address the barriers that are common among industries, such as lack of breaks, lack of food availability, lack of healthy options, and impacts of altered eating timing on shiftworkers. Base guidelines could then be tailored for specific industries to meet the industry specific barriers that were also identified in the comments in this study. 

Partners of shiftworkers are represented in the current study, and this presents a new perspective on the eating patterns of workers. While previous literature has explored the impact of shiftwork on partners and families of shiftworkers and their view on the shift schedule [48,49,50], no research has explored how the eating behaviour of shiftworkers is influenced by their partner, spouse, or family. In the current study, spouses of shiftworkers talked about cooking and packing food for their shiftworking partner. This is important to consider when providing guidelines and recommendations to shiftworkers, as it may not be the workers themselves that would action information about what to bring on-shift. Further, as these comments were largely from shiftwork related Facebook pages, it is clear that family members are engaged in shiftwork communities, and should be engaged in research aiming to understand the life and family context of shiftworkers. 

Understanding and optimizing the timing of eating for shiftworkers is an established research need [22]. Strategies for scheduled eating, such as TRE and IF are popular amongst the general population and have been shown to be effective for weight loss and cardiometabolic health [31,51,52,53,54]; investigating the effectiveness and feasibility of these strategies in shiftworking populations is an emerging research area [22,55]. Findings of the current study suggest that altered eating schedules and not knowing when to eat is a common problem experienced by shiftworkers and thus, scheduled eating strategies may be useful in this population. However, this study is the first to report attitudes that shiftworkers have to these eating schedules, and to report the experiences of workers who have been trying these schedules already. Comments from some shiftworkers expressed that using a TRE schedule has led to weight loss. This supports the findings of a recent study by Manoogian et al. [56], suggesting that not only was TRE feasible for the sample of firefighters, but it improved cardiometabolic health [56]. However, comments from our study also demonstrated that some workers had difficulty with TRE when scheduling set eating timing around irregular shift times. This highlights the need to continue investigating TRE strategies in shiftworkers with different shift-types, in addition to continuing to explore feasibility, and mental and physical health outcomes. Intermittent fasting was only minimally represented in the comments of the current study, with several describing fasting as doable due to minimal time to eat on shift given their work schedule. Compared to TRE, IF has been less explored in shiftworkers, with one study currently underway [55]. While there were positive attitudes towards IF in the current study, there is emerging evidence that promoting fasting strategies could encourage disordered eating [57,58,59]. Therefore, IF should also be researched in the future in shiftworkers, in addition to TRE, with a particular focus on psychological health and wellbeing.

This study is the first to explore the eating behaviours of shiftworkers using Facebook comments on public pages. This novel methodology is a strength of the study as it allows for unbiased and exploratory data collection from shiftworkers from a variety of industries and with varying shiftwork experiences. However, there are several limitations of this study to consider when interpreting the findings. Facebook does not have functionality for Boolean search terms to search for public pages, public groups, and public comments, which therefore limits how systematic the search strategy could be. As data saturation was reached, we are confident we identified the key themes available from the comments. A further limitation of the methodology is that because of the nature of public domain data and keeping the identity of the authors of the comments anonymous, we were unable to collect detailed demographic information. We therefore do not know the representation of sex, age, cultural background, years working shiftwork, industry and other variables that would add to the understanding of the results. However, this study has provided useful information about key issues that are relevant for shiftworkers, and future research can build on this research in qualitative and quantitative studies with methodology that allows for demographic information to be collected and an equal representation of different groups. Lastly, only comments written in English were included in this study which likely means the shiftworkers sampled were from English-speaking backgrounds. There are cultural differences in the eating patterns of shiftworkers [21,26] and only including comments written in English may have limited the representation of these differences. Future research should be mindful of this and sample comments from a range of languages. 

## 5. Conclusions

The current study is the first to explore the eating behaviours of shiftworkers through comments on Facebook. Findings demonstrate common experiences and barriers to healthy eating behaviours faced by shiftworkers across different industries, which suggests the importance of developing general guidelines and recommendations for shiftworker eating patterns that can then be tailored for individuals as needed. Given the link between eating patterns on-shift and the risk of long-term health issues for shiftworkers, creating targeted recommendations that address the issues described by shiftworkers is of utmost importance. This will ultimately give rise to shiftworkers who are empowered to optimise their eating behaviours. 

## Figures and Tables

**Table 1:** Code Characteristics and Theme Descriptions.

Categories Used in Deductive Coding (Based on [26])	Number of Comments (%)	Themes
What type of foods do shiftworkers eat?	74 (51)	Unhealthy foods used to cope with sleepiness on shift. Workplace culture influencing food choice. Difficulty following healthy eating plans.
Where is food sourced from?	38 (26)	Limited access to external food sources to for purchasing healthy foods. Difficulty with storage of healthy foods.
When do shiftworkers eat?	36 (25)	Break schedule influences when food can be eaten. Job demand influences when food can be eaten. Scheduled eating practices (e.g., TRE) in a shiftwork setting
Why do shiftworkers choose to eat on shift?	51(35)	Choosing healthy or unhealthy foods to improve alertness and productivity. Weight changes due to unhealthy foods. Gastric upset influencing the choice to eat healthy or unhealthy foods.

Note: Comments could be categorised into multiple codes or themes. TRE: Time-Restricted Eating.

## Data Availability

Data available on request.
